# Metabolomic Study on the Preventive Effect of *Patrinia scabiosaefolia* Fisch on Multipathogen Induced Pelvic Inflammatory Disease in Rats

**DOI:** 10.1155/2015/170792

**Published:** 2015-06-14

**Authors:** Wei Zou, Xiaoke Wen, Yi Zheng, Zuoqi Xiao, Jieying Luo, Shuqiong Chen, Yichao Wang, Zeneng Cheng, Daxiong Xiang, Yichu Nie

**Affiliations:** ^1^The Maternal and Child Health Hospital of Hunan Province, Changsha 410008, China; ^2^Key Laboratory of Hunan Province for Traditional Chinese Medicine in Obstetrics & Gynecology Research, The Maternal and Child Health Hospital of Hunan Province, Changsha 410008, China; ^3^School of Pharmaceutical Sciences, Central South University, Changsha 410011, China; ^4^College of Pharmacy, Hunan University of Chinese Medicine, Changsha 410007, China; ^5^Clinic Pharmacy Research Laboratory, Second Xiangya Hospital of Central South University, Changsha 410011, China; ^6^State Key Laboratory of Respiratory Diseases, The First Affiliated Hospital of Guangzhou Medical University, Guangzhou 510120, China

## Abstract

*Patrinia scabiosaefolia* Fisch (PSF), a well-known traditional Chinese medicine (TCM), has been used as a “heat-clearing and detoxifying” agent. The present study was to illustrate the preventive effect of PSF on pelvic inflammatory disease (PID) in rats. The PID model was constructed by multipathogen infection of the upper genital tract with reference to the method previously reported. Urine metabolomic analysis was conducted with a GC-MS coupled with derivatization method. In this study, PID rats showed obvious infiltration of inflammatory cells and elevated expression of cytokines (IL-1*β* and IL-6) in upper genital tract, compared with control rats. Sixteen differentiating metabolites contributed to the alteration of metabolic profile in PID rats, including two amino acids, three fat acids, nine organic acids, and two types of sugars. The rats, infected by multipathogen and administered with PSF, showed decreased infiltration of inflammatory cells and lowered expression of cytokines in upper genital tract, compared with PID rats. Meanwhile, PSF intervened in the PID-associated alterations in TCA cycle, sugar metabolism, amino acid metabolism, and other uncertain metabolic pathways. These results indicate that PSF has preventive effect on multipathogen induced PID and holistic interventional effect on disease-associated metabolomic change.

## 1. Introduction

Traditional Chinese medicine (TCM), a complementary and alternative medicine, is considered to be a holistic approach that attempts to bring the body, mind, and spirit back to harmony [1]. As a part of system biology, metabolomics focuses on comprehensive determinations of all endogenous low molecular weight metabolites* in vivo*, and the change of metabolite profile can present the pathological or physiological change in holistic context from the metabolism aspect [2, 3]. Therefore, metabolomics meets the requirement of holistic characteristics of TCM, provides an opportunity to scientifically express the meaning of evidence-based Chinese medicine, and reduces the gap between TCM and modern drug discovery demand [1]. Thus, increasing studies in the fields of pharmacology [4, 5], toxicology [6], and pharmacokinetics [7] of TCM have resorted to metabolomics methods.

Pelvic inflammatory disease (PID) includes endocervicitis, endometritis, salpingitis, and peritonitis, caused by ascending infection of the upper female genital tract [8]. This disease has been considered to be a threat to women as it can lead to serious long term sequelae, such as tubal factor infertility, ectopic pregnancy, and chronic pelvic pain. The common pathogens of PID include genital mycoplasmas, gram-negative bacteria, gram-positive bacteria,* Chlamydia trachomatis*, and* Neisseria gonorrhoeae* [9–11]. Antibiotic regimen is the current strategies for conventional PID treatment [8], but the long term use of antibiotics will lead to antibiotic resistance. Therefore, complementary and alternative medicine could be a good choice for PID treatment.

*Patrinia scabiosaefolia* Fisch (PSF), a well-known TCM, is clinically used as a “heat-clearing and detoxifying” agent. Several chemical components of PSF have been identified, including triterpenes [12, 13], iridoids [14], saponins [15, 16], and lactones [17]. PSF showed multiple bioactivities, such as inhibiting colorectal cancer [18, 19], promoting the apoptosis of human multiple myeloma cells [20] and breast carcinoma MCF-7 cells [21], sedative, hypnotic [22], antiulcerative colitis [23], antipancreatitis [24], anti-inflammatory in RAW 264.7 cells [25], and antibacteria activity [26]. Nowadays, PSF is usually prescribed as a constituent in TCM prescriptions used for PID treatment [27, 28]. However, the pharmacological effect of PSF against PID* in vivo* is still unclear. The present study aims to explore the preventive effect of PSF on PID in a rat model and further evaluates its pharmacological effect through metabolomic research.

## 2. Materials and Methods

### 2.1. Reagents and Materials

Progesterone injection was purchased from Xianju Pharma (Taizhou, China). Pentobarbital was obtained from Xiya Reagent (Chengdu, China). Ultrapure water was produced by a Milli-Q plus water purification system (Milford, USA). N,O-bis(trimethylsilyl)-trifluoroacetamide-trimethylchlorosilane (BSTFA-TMCS) (99 : 1, v/v), urease, margaric acid, tropic acid, methoxylamine hydrochloride, pyridine, and metabolite standards were purchased from Sigma-Aldrich (St. Louis, USA). Absorbable gelatin sponge was purchased from Jinling Pharmaceutical Co. (Nanjing, China).

PSF was purchased from Tianxiang Co. (Yueyang, China) and identified by Professor Zhuxin Wang (Hunan university of Chinese medicine, Changsha, China). A voucher specimen (number HHBJC20140913) is deposited in the Key Laboratory of Hunan Province for Traditional Chinese Medicine in Obstetrics & Gynecology Research (the Maternal and Child Health Hospital of Hunan Province, Changsha, China). Referring to previous method [25], a 500 g of PSF was two times extracted with 5 L of hot ethanol for 1 hour and then the two extracts were filtrated. The filtrate was dried* in vacuo* to yield 63.9 g of the PSF extract.

### 2.2. Animals

Female specific pathogen-free (SPF) Sprague Dawley (SD) rats, 9-week aged and weighing 210–230 g, were purchased from Silaikejingda Experimental Animal Co. (Changsha, China). Standard temperature conditions (22°C) and a 12 h light/dark cycle were kept, and standard laboratory chow was available* ad libitum* through the whole experiment. The experimental procedures were approved by the Animal Care and Use Committee of the Central South University.

### 2.3. PID Model and Drug Administration

Twenty-four rats were randomly divided into control group, PID group, and PSF group, acclimated for a week and then injected subcutaneously with 10 mg progesterone a week before infection. The construction of PID model referred to the method previously reported [29, 30]. The pathogenic* Escherichia coli* strain (number 0573) and* Ureaplasma urealyticum* strain (t-strain mycoplasma) were obtained from Clinical Laboratory Department of the Maternal and Child Health Hospital of Hunan Province. Gelatin sponges, with volumes of 0.125 mL, were saturated with microbe-mixing solution with* U. urealyticum* concentration of 1 × 10^8^ ccu/mL and* E. coli* concentration of 1 × 10^8^ cfu/mL. The microbe-containing gelatin sponge was placed in each cervix of the rat in PID or PSF group, and then the rats were made to stand upside down for 3 minutes. The cervixes of control group rats were implanted with gelatin sponges saturated with saline. This infection procedure was conducted once every two days and repeated four times. Surface disinfection was conducted with 70% alcohol after each infection. From the first infection, PSF group rats were orally administered with PSF extract at dose of 600 mg/kg/day which is equivalent to the clinical dose of approximate 830 mg/kg/day of PSF, and rats in PID or control group were gavaged with the same volume of saline. On the 21st day, the 24 h urine of each rat was collected and restored at −80°C. Then, vaginal swab samples were obtained to detect the* U. urealyticum* and* E. coli* by using mycoplasma detection kit and TDR-300B automatic microbe analysis system (Tiandiren, Changsha, China), respectively. After that, rats were anesthetized with pentobarbital (with a dose of 30 mg/kg). Plasma and the right upper genital tract (including uterus and fallopian tube) were collected and restored at −80°C, and the left upper genital tract was used for histological examination, followed by cervical dislocation of all the rats.

### 2.4. Histological Examination

The left uterus and fallopian tube of each rat were used to conduct histological examination with hematoxylin and eosin (HE) stain. Three different parts for each tissue sample were examined under light microscopy (×100) by a blinded observer. Then, the observer semiscored the inflammation in uterus and fallopian tube on degree of inflammatory cells infiltration (graded from 0 to 3).

### 2.5. Examinations of C-Reactive Protein (CRP), Interleukin-1*β* (IL-1*β*), and IL-6

Enzyme-linked immunosorbent assay (ELISA) was conducted to determine the concentration of CRP in plasma by kits from Raybiotech (Norcross, USA). The right upper genital tract (including uterus and fallopian tube) of each rat was added to physiologic saline at the ratio of 1 : 5 (w/v), followed by homogenization. The amount of homogenate protein was measured with BCA assay kit (Beyotime, Shanghai, China). The concentrations of IL-1*β* and IL-6 in homogenate were determined by ELISA kits (Raybiotech, Norcross, USA) and presented as *μ*g/g protein of homogenate.

### 2.6. Urine Sampling

Urine samples were diluted to give a creatinine concentration of 2.5 mmol/L, which was determined by a JEOL JCA-BM1650 clinical biochemistry analyzer (Tokyo, Japan). Thirty units of urease were added into 100 *μ*L of urine, followed by incubation for 30 min at 37°C. Then, 25 *μ*g margaric acid and 25 *μ*g tropic acid were added as internal standards. An aliquot of 800 *μ*L of ethanol was mixed with sample, and then the mixture was centrifuged at 12,000 g for 15 min. The supernatant was transferred to a new tube and stored at −20°C for 10 min. The supernatant was dried in a vacuum dryer at room temperature, and 100 *μ*L of methoxylamine hydrochloride (dissolved in pyridine) with a concentration of 20 mg/mL was added, mixed, and incubated at 37°C for 2 h. Then, a 100 *μ*L of BSTFA-TMCS was added and incubated at 70°C for 1 h. Quality control (QC) samples were prepared with the same procedure.

### 2.7. GC-MS Conditions

GC-MS analysis was conducted on an Agilent 7890 GC system coupled with an Agilent 5975C mass analyzer (Agilent, Palo Alto, USA). Chromatographic separation was carried on an Agilent DB-5MS capillary column (30 m × 0.25 mm ID × 0.25 *μ*m film thickness). One microliter of the derivatized sample or reference standard was injected in the splitless mode. Helium was used as carrier gas with flow rate of 1.5 mL/min. The temperatures of inlet, transfer line, and ion source were maintained at 250, 300, and 230°C, respectively. The GC temperature programming was set to 4 min isothermal heating at 60°C, followed by the first ramp at 6°C/min to 150°C and holding for 8 min, second ramp at 8°C/min to 280°C and holding for 10 min, and third ramp at 12°C/min to 320°C. Mass was in the electron impact mode at 70 eV and in the full-scan monitoring mode from* m/z* 35 to 750. The 1701EA station software (Agilent, Palo Alto, USA) was used to acquire chromatogram and detect mass spectral peaks.

### 2.8. Data Processing, Multivariate Data Analysis, and Differentiating Metabolite Identification

In each sample, peak areas of metabolites were normalized by the peak area of the creatinine. Internal standards were used to calibrate the retention time of all metabolites and monitor sampling and instrumental condition. A data set of all samples, consisting of the retention time and the normalized peak area of metabolites, was imported into SIMCA-P+ software (version 11, Umetrics, Umeå, Sweden) for multivariate analysis. The data were processed by unit variance scaling and were mean-centered, followed by multivariate analysis including principle component analysis (PCA) and orthogonal signal correction filtered partial least squares discriminant analysis (OPLS-DA). PCA, the unsupervised statistical analysis, was used to describe associations and patterns among a set of variables. R2X and Q2 are two indexes of PCA model quality. Metabolites significantly contributing to group discrimination were selected by the values of variable importance in the projections (VIP > 1) through OPLS-DA. The independent-samples* t*-test was used to check the differentiating metabolites whose VIP > 1, and the differentiating metabolites were selected when *P* < 0.05. Presumable identification of their chemical structures was conducted by comparing their mass fragmentation patterns with the NIST/EPA/NIH Mass Spectral Library 2011 (NIST11, Gaithersburg, MD, USA). The further identification was performed by comparing their mass spectra and chromatographic retention times to standards.

### 2.9. Statistical Analysis

Statistical analysis was performed by an independent-samples* t*-test or one-way ANOVA followed by post hoc Dunnett T3 test in SPSS software (version 16.0, Chicago, USA). For all experiments, *P* < 0.05 was taken as statistically significant. Data were presented as mean ± standard deviation (SD).

## 3. Results

### 3.1. Pathogens and Histological Examination

**Figure 1:**
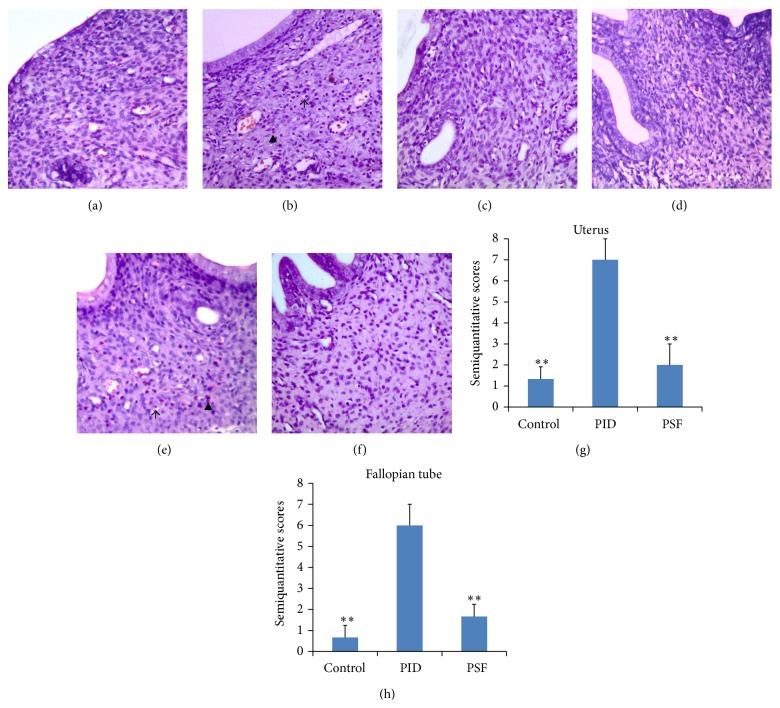
Histological changes in uterus and fallopian tube after pathogen infections and administration of PSF extract. Representative micrographs of uterus and fallopian tube stained by H & E (×100) are showed: (a) uterus of control group; (b) uterus of PID group; (c) uterus of PSF group; (d) fallopian tube of control group; (e) fallopian tube of PID group; and (f) fallopian tube of PSF group. The positive staining of neutrophil is indicated as ▲, and the lymphocyte cell infiltration is indicated as ↑. Histological semiquantitative scores of inflammatory cells infiltration in uterus (g) and fallopian tube (h) are presented. Each bar represents the mean ± SD (^∗∗^ *P* < 0.01, significantly different when compared with PID group; *n* = 3).

The pathogen examination of vaginal swab samples from PID rats showed positive for both* U. urealyticum* and* E. coli*, but the samples from control rats showed negative results. These results indicated successful infection in PID rats. The vaginal swab samples of PSF group showed negative for both pathogens, suggesting the antimicrobe effect of PSF extract* in vivo*. Compared with control rats, PID rats showed obvious uterus (Figure 1(b)) and fallopian tube (Figure 1(e)) inflammation, including mass neutrophil and lymphocyte infiltration. In PSF group, the inflammatory cells infiltration in upper genital tract was obviously decreased compared with PID group (Figures 1(c) and 1(f)). Semiquantitative scoring on uterus (Figure 1(g)) and fallopian tube (Figure 1(h)) inflammation showed the significant differences between PID group and control group. The score of PSF group was significantly lower than that of PID group, indicating the effect of PSF extract on decreasing inflammatory cells infiltration after pathogens infection.

### 3.2. Determinations of C-Reactive Protein (CRP), Interleukin-1*β* (IL-1*β*), and IL-6

As presented in Table 1, PID rats showed significantly higher level of CRP in plasma, compared with control and PSF group rats, and statistical difference was not observed between control group and PSF group. The concentrations of IL-1*β* and IL-6 in upper genital tract (including uterus and fallopian tube) of PID group rats were significantly higher than those of control and PSF group rats. These results indicated the inflammatory response in upper genital tract of PID rats and the anti-inflammatory effect of PSF extract.

### 3.3. Repeatability and Stability of the Analysis Method

**Figure 2:**
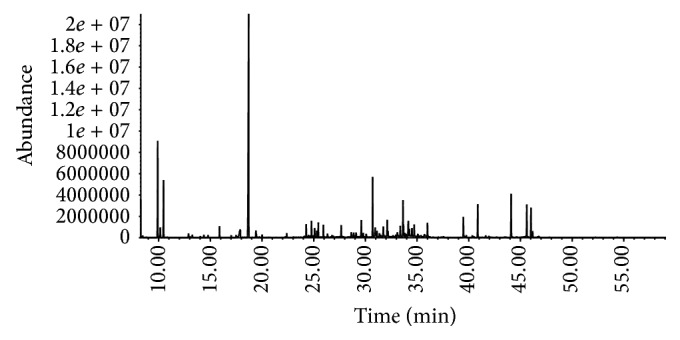
Representative base peak intensity chromatogram of the rat urine based on GC-MS.

By using the GC-MS coupled with derivatization method, representative chromatogram is showed in Figure 2. The stability and repeatability were evaluated by analyzing 6 injections of a QC sample and 6 QC samples, respectively. The RSDs of eight common ions in stability test were less than 0.23% for retention times and less than 4.63% for peak areas. The RSDs in repeatability test were less than 9.57% for peak areas. Therefore, the analytical method is repeatable and stable for metabolite determination.

### 3.4. Multivariate Statistical Analysis and Differentiating Metabolite Identification

**Figure 3:**
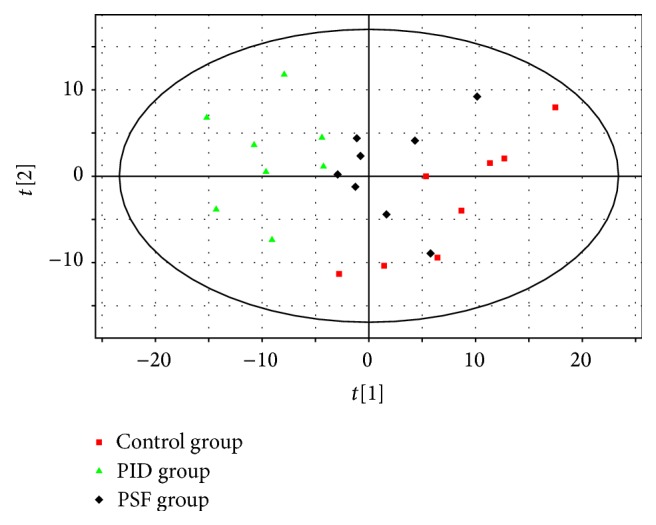
PCA scores plot based on the metabolomic data obtained from urine in 3 groups (R2X = 0.713, Q2 = 0.519; *n* = 8). The red squares indicate control group, the green triangles indicate PSF group, and the black diamonds indicate PID group.

**Figure 4:**
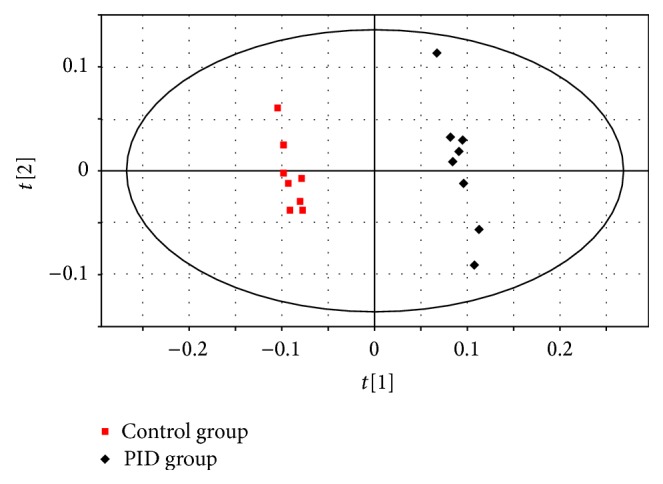
OPLS-DA scores plot based on the metabolomic data obtained from urine in control group and PID group (R2X = 0.605, R2Y = 0.996, Q2 = 0.978; *n* = 8). The red squares indicate control group, and the black diamonds indicate PID group.

**Figure 5:**
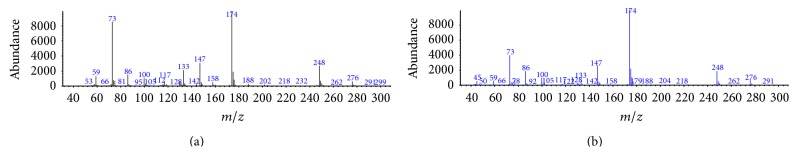
Representative mass spectrum of differentiating metabolite (a) and glycine standard (b).

A PCA model (with three components, R2X = 0.713 and Q2 = 0.519) was employed to reveal the separations between three groups. As showed in Figure 3, both control and PSF groups were clearly separated from PID group, and PSF group was partly overlapped by control group. It indicated that metabolomic change occurred in PID rats and PSF extract partly intervened in the change. An OPLS-DA model (with two components, R2X = 0.605, R2Y = 0.996, and Q2 = 0.978) showed a clearer separation between control group and PID group (Figure 4), and sixteen differentiating metabolites were selected with VIP > 1 and *P* < 0.05. Then, the chemical structures of these metabolites were presumed by comparing their mass fragmentation patterns with mass spectral library and then were identified by comparing their mass spectra and chromatographic retention times with that of standards. Typical mass spectrum of glycine in urine and standard are showed in Figure 5. The differentiating metabolites included pyruvate, succinate, 2-hydroxybutyrate, arachidonate, alanine, glycine, malate, glutarate, adipate, lactate, citraconate, citrate, acetate, aconitate, galactose, and glucose.

### 3.5. Effect of PSF Extract on Differentiating Metabolites Associated with PID

**Figure 6:**
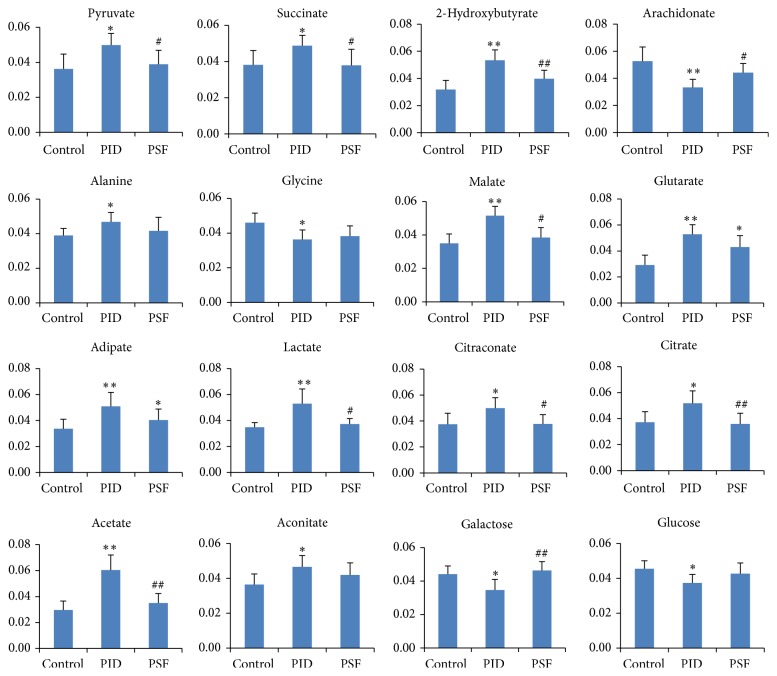
PID-associated differentiating metabolite changes after pathogen infections and administration of PSF extract. The value of the *y*-axis represents mean normalized peak areas of these metabolites. Each bar represents the mean ± SD (^∗^ *P* < 0.05, ^∗∗^ *P* < 0.01, significantly different from control group; ^#^ *P* < 0.05, ^##^ *P* < 0.01, significantly different from PID group; *n* = 8).

To further illuminate the interventional effect of PSF extract on the metabolomic change associated with PID, the normalized peak areas of sixteen differentiating metabolites in control group, PID group, and PSF group are showed in Figure 6. The levels of ten metabolites in PSF group were significantly different from PID group, and the concentrations of fourteen metabolites showed no statistical differences from control group. These results suggested that PSF extract prevented significant changes of most of PID-associated differentiating metabolites. However, the levels of glutarate and adipate in PID rats could not be statistically decreased by PSF extract.

## 4. Discussion

PSF is usually prescribed as one of the herbal materials in some TCM prescriptions which are used to treat PID [27, 28]. However, the pharmacological action of PSF in these TCM prescriptions is unclear. The present study aims to illuminate the preventive effect of PSF on PID in rat disease model.

Pathogens of PID could be recognized by Toll-like receptors (TLRs) in genital tract, which triggers inflammatory response [31].* U. urealyticum* and* E. coli* are two common PID pathogens [32]. Here, a mixed solution including* U. urealyticum *and* E. coli*, which could activate TLR 2 and TLR 4, respectively [33, 34], and hence induce an enhanced inflammatory response in the upper genital tract, was used to construct PID rat model according to the method reported previously [29, 30]. In the present pathogen examination, PSF extract showed antipathogen effect* in vivo*, which was consistent with its antimicrobe effect* in vitro* [26]. As the plasma CRP level has been included in the criteria for clinical diagnosis of PID [35], we also observed the concentration changes of CRP to illustrate the inflammation in PID group and anti-inflammatory effect of PSF extract. Elevated IL-1*β* in upper genital tract indicates the pathological development of PID [36], while increasing production of IL-6 is involved in the processes of chronization and can suppress the synthesis of IL-1*β* in the second phase of the immune response [37]. In our study, the concentrations of IL-1*β* and IL-6 in uterus and fallopian tube were significantly deceased in PSF group compared with PID group, indicating the anti-inflammatory effect of PSF extract. These results are consistent with the previous result that PSF inhibits expression of proinflammatory factors in RAW 264.7 cells* in vitro* [25].

TCM is a holistic strategy that attempts to bring the body back to harmony, and metabolomics could exhibit the holistic body condition. Therefore, a metabolomic study was conducted to better understand the pharmacological action of PSF in rats. In the present study, sixteen differentiating metabolites were found to be associated with PID. The biochemical reactions of these metabolites were obtained from the Kyoto Encyclopedia of Genes and Genomes (KEGG, http://www.genome.jp/kegg/) and the Human Metabolome Database (HMDB, http://www.hmdb.ca/). Our investigation revealed that PID-associated metabolism changes were involved in the citric acid cycle (TCA cycle), sugar metabolism, amino acid metabolism, fat acid metabolism, and other uncertain metabolic pathways. These differentiating metabolites may be potential biomarkers for diagnosis of PID, though further clinical experiments are needed to investigate their practical values.

Compared with control group, PID group showed the concurrence of decrease of types of sugars (glucose and galactose) and increase of pyruvate, suggesting an enhanced glycolysis in PID. Increase of pyruvate can enhance production of lactate, which was also observed in the present study. In PSF group, the types of sugars were increased, while pyruvate and lactate were decreased, indicating that PSF extract suppressed the PID-associated enhanced glycolysis. In PID group, the elevated intermediate products in TCA cycle, including citrate, succinate, aconitate, and malate, suggested possible defects in the mitochondrial respiratory system, and the changes of alanine and glycine levels demonstrated the alterations of amino acid metabolism. On the other hand, PSF extract showed the abilities of preventing mitochondrial respiratory system defects and intervening in PID-associated alterations of amino acid metabolism. The elevated fat acids (glutarate and adipate) and citraconate in PID rats suggested abnormalities of fat acid metabolism and 5-branched dibasic acid metabolism, respectively, which were also observed in arthritis [38, 39]. In this study, PSF extract had a positive effect on restoring citraconate level.

Leukotriene B4 (LTB4) and prostaglandin E2 (PGE2), which are products of arachidonate metabolism, exert important actions in histological changes in PID, such as leucocyte recruitment, oedema, and hyperalgesic response [40–42]. PSF extract suppressed inflammatory response and thus could lower the production of LTB4 and PGE2, which could be one of the reasons for the significantly elevated level of arachidonate in PSF group compared with PID group. 2-Hydroxybutyrate is a by-product of ophthalmate synthesis, the high level of which indicates the glutathione depletion [43]. The increased 2-hydroxybutyrate in PID group suggested the shortage of glutathione and the existence of oxidative stress in genital tract, and the decreased 2-hydroxybutyrate in PSF group indicated the preventive effect of PSF extract on oxidative stress. The vaginal lubrication contains a certain amount of acetic acid which appears as an antibacterial agent. Interestingly, the elevated acetate was observed in PID group, as the redundant pyruvate could be metabolized by pyruvate dehydrogenase to promote the production of acetate. The elevation of acetate may be deemed as a physiological response to defend the next infection, though studies are needed for confirmation in the future. Here, PSF extract showed the ability of suppressing the increase of acetate associated with PID.

## 5. Conclusions

In this study, the preventive effect of PSF against PID was found in a rat model. A GC-MS coupled with derivatization method was used to determine the PID-associated metabolomic change in urine and to investigate the interventional action of PSF extract. Through OPLS-DA process, two amino acids, three fat acids, nine organic acids, and two types of sugars were selected as differentiating metabolites, which related to TCA cycle, sugar metabolism, amino acid metabolism, fat acid metabolism, and other uncertain metabolic pathways. PSF extract showed significant effect on intervening in most of PID-associated metabolism alterations.

## Figures and Tables

**Table 1:** Changes of CRP in plasma and IL-1*β* and IL-6 in upper genital tract after pathogen infections and administration of PSF extract.

	CRP (*μ*g/mL)	IL-1*β* (*μ*g/g)	IL-6 (*μ*g/g)
Control group	95.60 ± 12.97	7.44 ± 0.77	5.02 ± 0.92
PID group	154.93 ± 29.53^∗∗^	9.27 ± 1.11^∗∗^	8.66 ± 1.06^∗∗^
PSF group	107.81 ± 12.18^##^	7.97 ± 0.81^#^	4.68 ± 0.70^##^

^∗∗^ *P* < 0.01, significantly different when compared with control group; ^#^ *P* < 0.05, ^##^ *P* < 0.01, significantly different when compared with PID group; *n* = 8.

## Abbreviations

GC/MS: Gas chromatography-mass spectrometric
PID: Pelvic inflammatory disease
PSF: * Patrinia scabiosaefolia* Fisch
PCA: Principal component analysis
OPLS-DS: Orthogonal signal correction filtered partial least squares-discriminant analysis
TCA cycle: Citric acid cycle
BSTFA-TMCS: N,O-Bis(trimethylsilyl)-trifluoroacetamide-trimethylchlorosilane
CRP: C-reactive protein
IL: Interleukin
QC: Quality control
VIP: Variable importance in the projections
TLRs: Toll-like receptors
LTB4: Leukotriene B4
PGE2: Prostaglandin E2.
